# Differences in functional exercise capacity and pulmonary function tests between acute respiratory distress syndrome (ARDS) of pulmonary and extrapulmonary origin

**DOI:** 10.1186/2197-425X-3-S1-A665

**Published:** 2015-10-01

**Authors:** B Llorente Ruiz, E Alonso Peces, M Vázquez Mezquita, O Navarrete Isidoro, J Luján Varas, R Molina Montero, C Pintado Delgado

**Affiliations:** Hospital Universitario Príncipe de Asturias, Intensive Care Medicine, Madrid, Spain; Hospital Universitario Príncipe de Asturias, Respiratory Services, Madrid, Spain

## Introduction

Treatment of ARDS with low volume tidal ventilation has shown improvement in survival rates as compared with traditional high tidal volume ventilation strategy. Nevertheles, respiratory morbidity, including impaired pulmonary function with obstructive and restricitive patterns, decreased DLCO diffusion and reduced exercise capacity have been described in survivors.

## Objectives

The aim of this study was to determine the differences in pulmonary function and functional status between ARDSp and ADRSexp survivors, after treatment with low volume tidal ventilation strategy.

## Methods

A total of 46 patients without comorbidity and more than 2-year survival after an ADRS episode were identified between 2000 and 2006. 27 patients finally participated in this study (13 ADRSp and 14 ADRSexp). Demographic data, smoke status, ventilator data, length of ICU and hospital stays and measures of severity of illness such as the Acute Physiology, Age, and Chronic Health Evaluation; Acute Lung Injury Score and Sepsis-Related Organ Failure Assessment were evaluated. Pulmonary function tests were performed using standard procedures. Spirometry, static volumes by pletismography and diffusing capacity form carbon monoxide by single-breath were measured. Normal values were calculated following the European Coal and Steel Union guidelines. Functional capacity was measure with a standardized six-minute walk test (6MWT) using standard protocol. Normal values for 6MWT were according with the *Enright y Sherill* reference equations.

## Results

There were no statistical differences in demographic and clinical variables analyzed, except for the number of days of ventilation treatment (25,38 ± 17,85 vs. 12,71 ± 10,00; p 0,030) and LIS at admission (3,06 ± 0,63 vs. 2,36 ± 0,99; p 0,038).

The lung function test values did not show statistical differences either, except for diffusion data, both DLCO (66,82 ± 16,95 vs. 81,88 ± 10,05, p 0,018) and KCO values (82,81 ± 14,98 vs. 98,54 ± 20,27,

p 0,031).

In the ARDSp group, 3 patients were unable to perform completely the 6MWT; all patients from the ADRSexp group perform it. 40% of the patient from the ARDSp group walked a distance lower than the predicted value in contrast with 7,14% in the ARDSexp group (p 0,025).

## Conclusions

A high percentage of previously healthy patients that survived an ARDS, presented changes in the lung function tests and reduced exercise capacity, even after two years of the clinical episode. There were no differences in long-term respiratory outcomes between ARDSp an ARDSexp, except for DLCO and KCO, that showed lower values in ARDSp group. ARDSp showed worse exercise capacity compared with the ARDSp group.
